# A conceptual framework for Emergency department design in a pandemic

**DOI:** 10.1186/s13049-020-00809-7

**Published:** 2020-12-17

**Authors:** Gayathri Devi Nadarajan, Eunizar Omar, Benjamin S. Abella, Pei Shan Hoe, Sang Do Shin, Matthew Huei-Ming Ma, Marcus Eng Hock Ong

**Affiliations:** 1 Department of Emergency Medicine, Singapore General Hospital, 1 Outram Road, Singapore City, 169608 Singapore; 2 Department of Emergency Medicine, Sengkang General Hospital, Sengkang, Singapore; 3 Center for Resuscitation Science and Department of Emergency Medicine, University of Pennsylvania, Philadelphia, USA; 4 Duke-NUS Graduate Medical School, Singapore City, Singapore; 5 Department of Emergency Medicine, Seoul National University College of Medicine, Seoul, South Korea; 6 Department of Emergency Medicine, National Taiwan University Hospital, Taipei City, Taiwan; 7 Department of Emergency Medicine, National Taiwan University Hospital Yunlin Branch, Douliou City, Taiwan; 8 Health Services and Systems Research (HSSR), Duke-NUS Medical School, Singapore City, Singapore

**Keywords:** SARS-CoV-2, COVID-19, Emergency department, Operation, Pandemic, Framework

## Abstract

**Background:**

The current COVID-19 pandemic is highlighting gaps around the world in the design and workflow of Emergency Departments (ED). These gaps have an impact on both patient care and staff safety and represent a risk to public health. There is a need for a conceptual framework to guide ED design and workflow to address these challenges. Such a framework is important as the ED environment will always remain vulnerable to infectious diseases outbreaks in the future.

**Aims:**

This paper aims to address issues and principles around ED design and workflow amidst the COVID-19 pandemic. We propose a conceptual framework and checklist for EDs to be prepared for future outbreaks as well.

**Methods:**

A scoping literature review was conducted, of the experiences of EDs in managing outbreaks such as SARS, H1N1 and COVID-19. The combined experiences of the authors and the experiences from the literature were grouped under common themes to develop the conceptual framework.

**Results:**

Four key principles were derived- (1) situational awareness, surveillance and perimeter defence, (2) ED staff protection, (3) surge capacity management and (4) ED recovery. The findings were integrated in a proposed conceptual framework to guide ED design in response to an infectious disease outbreak. There are various elements which need to be considered at ED input, throughput and output. These elements can be categorised into (1) ***system*** (workflow, protocols and communication), (2) ***staff*** (human resources), (3) ***space*** (infrastructure***)***, and (4) ***supply*** (logistics) and are placed in a checklist for pragmatic use.

**Conclusion:**

The ED needs to be in a constant state of preparedness. A framework can be useful to guide ED design and workflow to achieve this. As all ED systems are different with varying capabilities, our framework may help EDs across the world prepare for infectious disease outbreaks.

## Background

We are currently in the midst of a global pandemic that has brought much of the world to a standstill. With containment efforts which left nearly one-third [1, 2] of the world’s population in lockdown, this public health emergency also poses a significant threat to global industries and economies [3]. After rapid spread following reports of the first cases in Wuhan, China in December 2019, the World Health Organisation (WHO) [4] declared this outbreak a public health emergency on 30th January 2020 and a pandemic on 11th March 2020. The death toll is increasing, and a tragic number of healthcare workers have lost their lives in the line of their duty, including those in the frontline from the Emergency Care Services (ECS).

ECS personnel are usually the first to recognise and respond to a disease outbreak, often prior to public health attention. The Emergency Department (ED) may thus receive the index cases of a new highly infectious disease. Failure to recognise the index cases and delays in management of an outbreak can pose global health risks [5] and risks to the health of medical staff [6].

The Severe Acute Respiratory Syndrome (SARS) outbreak in 2003 represented a dramatic learning experience for the Emergency Medicine community [7–10]. It highlighted the vulnerability of the ED environment and stressed the importance of ED design, healthcare staff protection and risk mitigation measures in infectious disease outbreak preparedness [11]. In Asia, it led to major changes in the design of EDs, birthing concepts such as pre-triage screening and designated “fever” units. SARS was a preview to its successor, the SARS-CoV-2 virus or COVID-19 which is now serving as a global reminder of the importance of ED design and workflow structure.

An infectious diseases outbreak is a global health security threat, similar to a natural disaster or bioterrorism attack. A robust risk mitigation system is paramount for EDs [12], which need to be in a state of constant readiness to respond to potential large-scale outbreaks. However, despite previous epidemics, there appear to be many gaps in ECS [13–15], which have an impact on health systems as seen with the current COVID-19 pandemic [4]. Personal Protective Equipment (PPE) shortages, nosocomial spread, and variable internal communication strategies represent examples of these gaps. Emergency frontline staff, patients and the general public will either be the beneficiaries of a well-prepared system or victims of poor planning.

### Current gap in literature

Although individual ED experiences during infectious disease outbreaks have been described in the literature [16–22], few works have outlined a pragmatic framework incorporating sound principles to guide ED design and workflow adaptations. This paper aims to address issues around ED design and workflow amidst the COVID-19 pandemic. We propose a conceptual framework and checklist for EDs to be ready for future outbreaks without compromising care of the broader patient population needing the ED.

## Methods

A scoping literature review was conducted looking at the experiences of various EDs [23–25] and adaptations made in managing outbreaks such as SARS, H1N1 and COVID-19 [26]. Pubmed, Proquest and Google Scholar were used to search up till the period of April 2020, during the peak of COVID and when the article was written. These search platforms cover a comprehensive range of COVID articles. The search string “Pandemic AND Emergency Department AND (“model” OR “management” OR “template” OR “framework”)” was used to look for existing frameworks, which yielded 1012 results. To look for the experiences of various ED with COVID-19, two search strings were used- “Pandemic AND Hospital AND (“model” OR “management” OR “template” OR “framework”) AND (“sars” OR “H1N1” OR “COVID”)” which yielded 3856 results. Proquest yielded a wider net of results for both, which included results from Pubmed.

Following which, 2 independent reviewers, reviewed the abstracts and selected the articles. Once the articles were selected, 2 Emergency Physicians (EP) (GD and EO) independently read through the articles to consolidate the content into common themes. The authors also shared their experiences from the different countries where they practice. The literature content and experiences were grouped into themes. These themes were verified by a third EP (MO) and two physicians (GD and MO) developed the conceptual framework. This conceptual framework was then discussed, revised and finalised by all the authors of the paper.

## Results

Four key principles guiding ED responses to an outbreak were identified. The principles described in more detail below make up the basis of our proposed conceptual framework.

### Situational awareness, surveillance and perimeter defence

Situational awareness and a surveillance system are paramount in identifying and isolating the first suspected cases and being alerted to new clusters. In a busy ED environment, early identification of an outbreak or cluster pattern is a major challenge. A good surveillance system needs to be in place even during non-outbreak periods.

Perimeter defence refers to the formation of a conceptual “perimeter” through ED design and workflow to segregate potentially infected patients from other non-suspect cases. This is a key containment measure as this protects the ED, hospital, staff and patients. Two spatial control strategies can be employed (defensive isolation versus offensive containment [27]) depending on the stage of the outbreak and the load of infectious suspects.

### Frontline staff protection

One of our top priorities is to advocate for zero infection amongst healthcare workers [23]. This can be done in several ways. First, there should be adequate PPE supply and appropriate training on their use. Risk-adapted use of PPE should be part of routine ED practice when managing any infectious diseases like chicken pox or tuberculosis. In an outbreak situation, supplies need to be scaled up rapidly to meet the expected increase in PPE requirement. Second, roster modifications such as adaptation of modular shift schedules [28] can allow for effective contact tracing and mitigate the risk of widespread nosocomial infections. Lastly, institutions should also have the capability to screen and test staff for illness and if available, provide them with antiviral prophylaxis and vaccines in view of the inherent risks they face in the line of duty.

### Surge capacity management

During a pandemic, ED caseload surges are inevitable. Surge capacity is the ability to manage patients above the usual volume [29] without compromising normal care. Surge capacity can potentially be increased by managing demand, establishing alternate care facilities, expanding bed capacity and minimizing resource consumption by patients [30]. The system needs resilience and flexibility to remain functional within the capacity of its available resources during such outbreaks [26].

### Ability to recover to its previous steady state

The ED needs to have a system in place to allow recovery to its usual steady state. This includes infrastructure as well as staff. Frontline staff need to be able to function at full capacity even after the outbreak period. At its recovery stage, ED volumes may even be higher due to the rebound of patients with complications from neglect of chronic conditions.

**Fig. 1:**
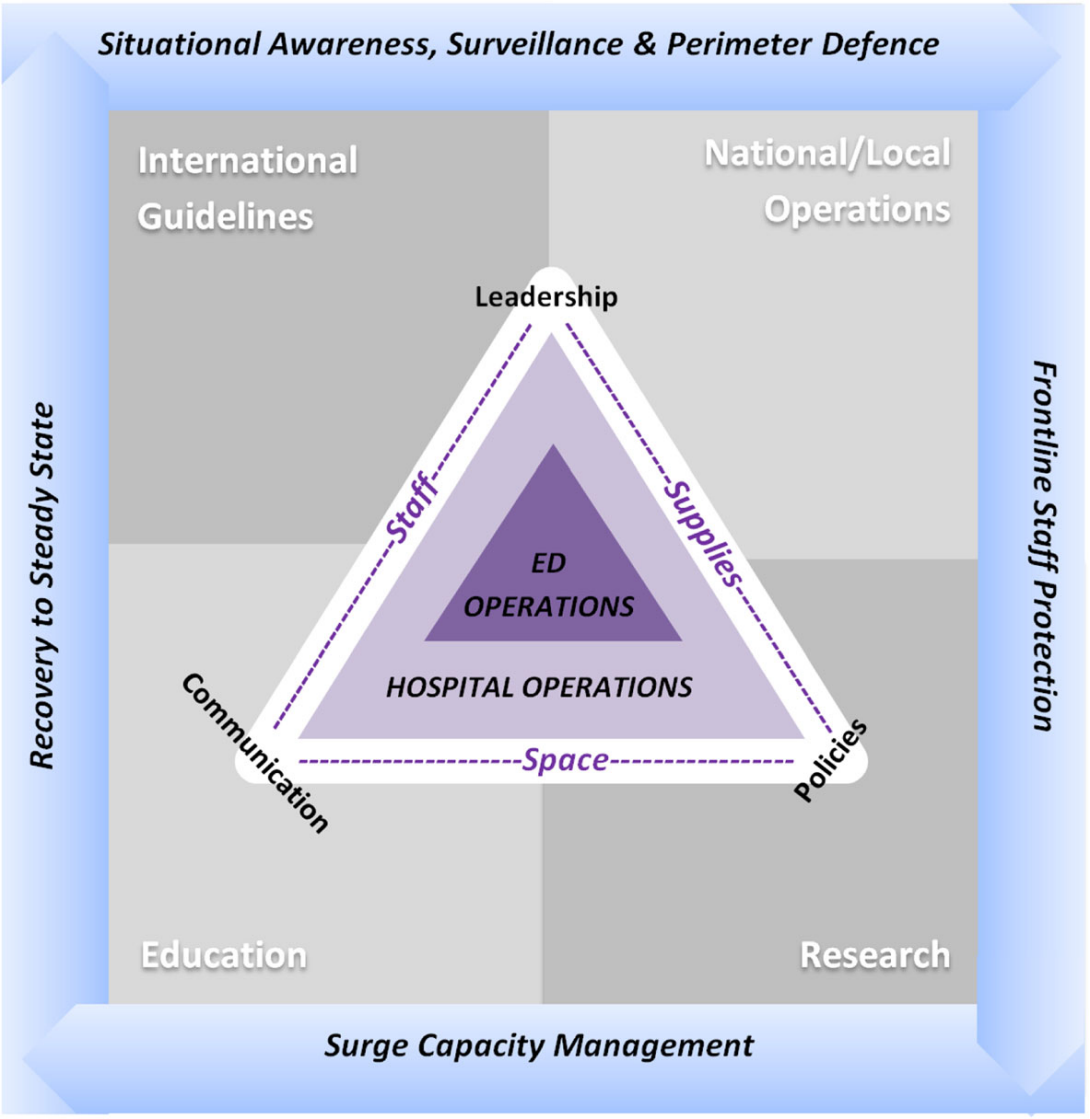
Conceptual framework describing relationship between state, hospital and ED in pandemic preparedness planning and response

Incorporating the principles above, we propose the following conceptual framework to depict this (Fig. 1). The principles form the border of our conceptual framework as they should govern international guidelines, national and local health system operations, research, public and staff education, and eventually the operations within the hospital and ED. International guidelines, health systems, research and education form the background that support hospital and ED operations, making way for cost effective policy implementation.

For the principles to be effectively implemented in the hospitals and ED, there needs to be good leadership, supporting policies and an effective flow of communication down the command line; hence forming the apices of the triangle [31].

The operations of the hospital and the ED are intertwined and limited by their resources as defined by space, staff and supplies. During a crisis, the perimeters of this triangle may be expanded (hence depicted by dotted lines); but is dependent on institutional leadership, policies and communication capabilities (as depicted by the apices of the triangle).

Space, staff and supplies from the hospital can be diverted to the ED to deal with the surge initially. Surge patients also need to be able to flow to infectious diseases units and intensive care units (ICUs) of the hospital. The following section describes a pragmatic approach within the ED workflow using the model above.

### ED input

#### Perimeter defence, situational awareness & surveillance

As the gateway to the hospital, perimeter defence is crucial within the ED. There can be two approaches to manage the entry of patients into the ED. Firstly, ED arrivals can be minimised through community-based screening/testing [18]. This reduces the need for relatively well patients to come to the ED as fever screening/testing could be better done in the community. The second approach is to appropriately isolate the suspect cases when they arrive in the ED, which will be discussed here.

Patients present to the ED triage via ambulance, referral from a primary healthcare facility or as walk-in. A pre-triage screening area is an important defensive isolation measure. Screening should be almost 100% sensitive [32] such that all suspect cases are separated from non-suspect cases. Screening criteria at the ED should be broader [32] than national or international guidelines [33, 34] to enable the identification of patients with atypical features, as seen with COVID-19. Early identification of COVID-19 cases in the ED itself is paramount as a single case managed in a non-isolated area by staff without appropriate PPE can result in nosocomial spread, quarantine of staff and disruption of hospital services [17].

This screening area can be located outdoors or within well-ventilated indoor areas, with an expandable space to allow for surges such that the risk of airborne transmission can be mitigated. Screening may even be done via telemedicine [21] or through a website [35] or call centre [18], which then channels patients to appropriate healthcare facilities (for systems where there is a designated infectious disease hospital).

Pre-hospital medical teams will need to work with the ED on an agreed workflow for the infectious risk status of patients with an unknown travel history or risk profile.

After screening, patients can be categorised into high, intermediate and non-suspect cases [8, 11, 25]. Rapid diagnostic test kits will facilitate the process and allow patients to be screened to “Confirmed” and “Non-suspect” categories. Without rapid diagnostic kits, the high and intermediate suspects would remain as Patients Under Investigation (PUI). Following this, the usual clinical triage process can take place to identify critically unwell patients who may need immediate attention.

We propose this screening process should be routine for all patients even during non-outbreak periods. For example, following the 2004 SARS outbreak, “fever zones” were set up in Singapore EDs, where patients with febrile illnesses and travel history to countries with suspected outbreaks are routinely isolated [11]. These zones are routinely staffed with healthcare workers donning appropriate personal protective equipment (PPE) and have negative pressure rooms to limit exposure to other patients and healthcare staff. Hence for COVID-19, when the index case presented to a tertiary hospital in Singapore, the patient was appropriately isolated [36].

Situational awareness is key to ensure accurate definition of suspect cases. When dealing with an outbreak of a novel pathogen such as SARS-CoV-2, screening criteria can change daily based on the latest developments both locally and globally. A robust link between epidemiological surveillance and ED workflow is required. Screening questions should remain dynamic and be updated regularly in tandem with surveillance data. For example, an ED surveillance team comprising ED staff with public health training serving as the liaison with the region’s public health officials may be useful [7]. Epidemiological surveillance [17] is important for identification of new clusters and symptomology of a novel disease.

The use of artificial Intelligence algorithms at the screening and triage area to pick up patterns of illness may also be helpful. (e.g.: patients’ location, symptom set). This surveillance system may be programmed to alert the ED when cluster patterns are noted [37]. During the outbreak itself, where there is augmented staff with a heterogeneous skill set, this system can help reduce cognitive load.

#### Frontline staff protection

In order to protect ED staff, a “no patient contact” method should be adopted at the screening area. In Singapore, self-service screening was piloted, where patients use a QR code on their phones to key their details into online forms which will automatically feed into patients’ electronic records.

Appropriate PPE for staff, such as the use of N95 masks, is also crucial during screening and triage. Patients should be given face masks and safe-distancing measures (e.g.: 1 m apart during COVID-19) should be enforced within patient waiting areas to prevent nosocomial spread.

There thus needs to be an emergency stockpile for PPE and disinfectant liquids for equipment and surfaces. Training and compliance to infection control measures need to be reinforced (such as handwashing and donning of PPE).

#### Surge capacity management

Surge plans for systems, space, supply and staff need to be activated once an outbreak is declared. The input into the ED may be reduced through early management of suspect cases within the community. For example, workflows can include designating primary healthcare facilities to perform screening. There can also be a central internet portal where the public can locate clinics that perform screening and their waiting times as well as clinics offering telemedicine services. Other options include “drive-through swabbing” [18, 38] or “walkthrough swabbing” [38] clinical sites.

The input within the ED can also be reduced by an “influenza clinic” [39] or assessment centre [40] outside the ED which rapidly sees suspect cases who can potentially be swabbed and discharged. In some countries such as the US, this often takes the form of tents in physical proximity to ED entrances.

There is a need to prepare for staff, space and supplies such that surge plans can be activated on short notice. Staff may be redeployed from other departments to augment manpower. The more experienced nursing staff should be placed at the triage area while augment staff can be designated to screening areas which have more standardised operating procedures.

## ED throughput

### Perimeter defence and situational awareness

Within the ED, patients should be seen and managed within their allocated areas (high, intermediate and non-suspect areas). There should be physical barriers between these allocated areas. Ideally the high suspect cases should be managed in isolated, negative pressure cubicles. The intermediate suspect can be managed as a cohort in a space which allows adequate (e.g.: 1 m) spacing between them. The use of radio-frequency identification (RFID) tags may be useful to allow tracking of the patients’ movements.

Visitor movements should be restricted, and policies made restricting visitation within the high or intermediate suspect areas. For this to occur, security should be activated and the ED entrances and exit areas should be cordoned off.

### Frontline staff protection

Staff working in a specific designated area for infected patients should ideally remain in that space throughout a work shift. If there is a need to travel to a different section, the full PPE should be changed. There should be clear instructions on the type of PPE to be worn in the respective areas. Disposable scrub sets and showering facilities within the ED are also important for staff protection.

In some systems, a modular staffing approach is adopted, such that one physician and nurse team remains in the high-risk suspect area for a specified period. Another type of modular staffing is a team-based, fixed shift system where the physicians and nurses work within the same team during the outbreak period to limit contact within the department [28]. If a team member is infected, the rest of the team members can be quarantined. This will also facilitate contact tracing when positive patients are seen by the team.

Aerosol generating procedures will need to be modified in a situation such as COVID-19, including reducing the use of nebulisers, non-invasive ventilation and even high flow oxygen. Appropriate equipment, PPE such as Powered Air-Purifying Respirator (PAPR) and a defined protocol needs to be in place for intubation procedures such that the staff involved are protected.

### Surge capacity

Within the three areas, there must be the capacity to manage patients of all clinical severity levels. Each area requires workflows for time sensitive conditions such as trauma, myocardial infarction or stroke. Relevant activations should occur for these cases, and the receiving specialist teams need to aware of the infectious profile of the patients (eg: high or intermediate suspect) so that the appropriate, timely disposition can be arranged.

Patients with suspicious complaints but who do not fall under the case definition criteria may have to be placed in the intermediate suspect area [41]. This will ensure that at the initial stages, when the disease transmission profile is unclear and the situation is dynamic, patients who could be potential suspects are placed away from non-suspects.

Consideration needs to be given to palliative or dying patients [21]. Many of them will present with respiratory symptoms. If they are seen in an intermediate or high-risk area, there should be a workflow to determine the number of family members allowed by their bedside and if possible, a separate, private room.

Some investigations will need to be expedited to reduce throughput times, for example swab results or chest x-ray reports. This will require a collaborative workflow with radiology and clinical laboratory departments. The ED can also limit and stratify relevant investigations to reduce strain on resources. The areas for investigations may need to be replicated for the high and low suspect areas such as availability of plain radiography capabilities in the high suspect areas.

Emergency observation units may have to cease operations or modified to make space and human resources available for the surge in patients.

## ED output

There needs to be a workflow to facilitate the disposition decision making process, specific to level of infectious concern. This will be hospital-specific depending on the hospitals’ resources. Disposition workflows should be dynamic as the outbreak situation evolves.

### Perimeter defence & situational awareness

If the investigations are not able to yield results during their stay in the ED, the stratification of high, intermediate and non-suspect must be maintained throughout, even at the output stage. In this situation, physical ward spaces should be in line with the ED suspect stratification system. For example, there may be a need for three different destination wards for the patients (e.g.: high suspect, intermediate suspect, non-suspect).

There should ideally be rapid movement of patients up to the ward. Hence, there needs to be close liaison with the inpatient wards and bed management unit (BMU). Delays are still to be expected and there should be an observation or in-patient bed waiting area within each of the suspect areas.

There should be workflows to help decide those who are safe to be swabbed and discharged. The ED can be designed such that those who are well enough to be swabbed and sent home, may not even need to enter the actual ED, as seen in Taiwan [42].

For those discharged, it is important to determine if they need to be on a stay home notice (SHN), and there should be an approach to perform contact tracing. Appropriate return advice should be given. The ED can work closely with the public health to enforce SHN by various methods (e.g.: RFID tagging, telemedicine).

### Frontline staff protection

After patient disposition is decided, patients may be moved to other areas to await transfer to beds or other isolation facilities. Proper hand-over between staff is essential. Designated, risk-adapted waiting areas also need to be available to maintain segregation of high suspect cases. Transfer of high suspect patients out of the ED itself can be challenging, as there is a risk of transmission en-route. In order to protect staff and passers-by from inadvertent exposure, the pathway for patient movement needs to be cleared, a task that may involve activating hospital security. If transfers to another facility is required, designated ambulance services should be available where transferring staff are aware of the risks and have proper PPE. Frontline staff protection also includes assigning dedicated decontamination (eg: showers), rest and recuperating areas. Having rest areas without a proper contamination area can be counterintuitive towards staff protection. If there are space constraints for a staff decontamination area, an alternative is to have a ‘clean’ and ‘dirty’ staff rest area. The system should also have a staff wellness or psychological first aid program in place to protect them from mental fatigue or burnout which are potential issues of such a pandemic.

### Surge capacity management

There needs to be a set of criteria to determine if patients require admission and the type of ward required (individual isolation for high suspect, cohort ward for intermediate suspect or normal ward for non-suspects). Close communication with the BMU is essential to ensure that patients are cleared quickly from the ED to accommodate for surges. Cancellation of elective admissions and expedited discharge of patients may need to take place to allow the flow of patients to the wards.

### Putting it together

**Fig. 2:**
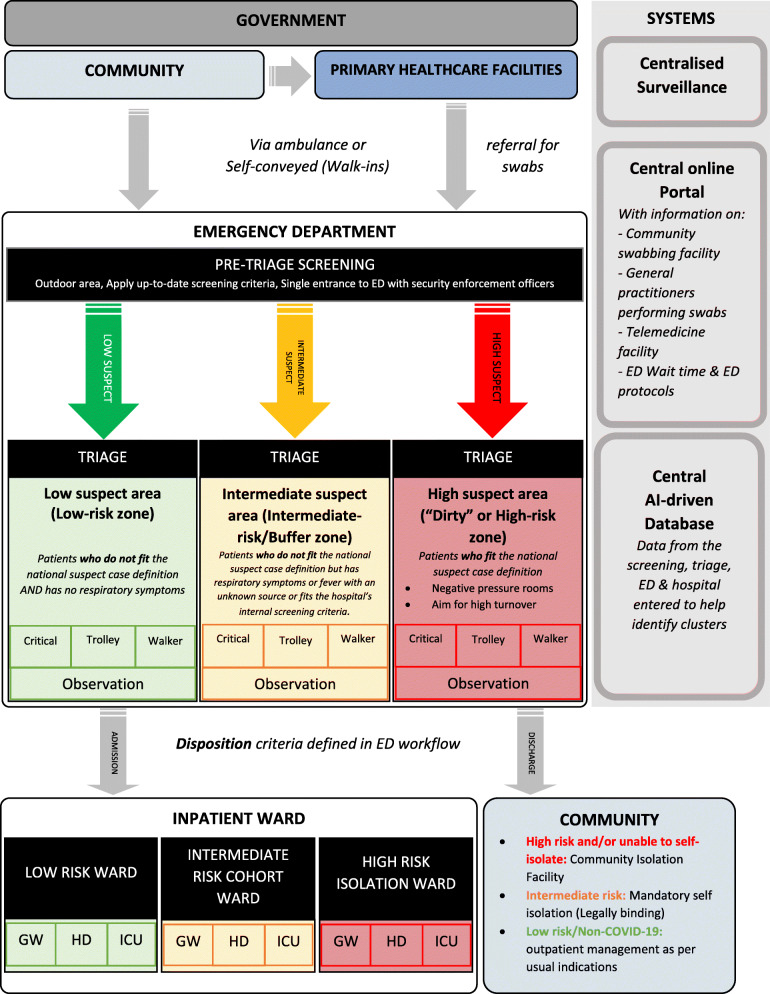
Healthcare system physical space and workflow model

From the key concepts identified above, we created a workflow model (Fig. 2) and checklist (Table 1) to guide ED design and workflow during COVID-19 as well as future outbreaks.

**Table 1:** Checklist for ED pandemic workflow

	Situational awareness & perimeter defence	ED staff protection	Surge capacity management
**ED Input**	SYSTEM		
	**Workflow & protocols**		
	□ Is there a single entrance into the ED? □ Is there a screening criterion for all ED patients? (incorporating clinical and epidemiological features) □ Is the screening criteria kept up-to-date? □ Are there clear triage criteria (high suspect, intermediate suspect & non-suspect)	□ Is there a protocol for the type of PPE to be worn by screening, triage and ancillary staff? □ Do all patients have to wear masks? □ Are there safe-distancing measures for patients in waiting areas? □ Is there a contactless screening system?	□ Is there a workflow to re-direct well patients to alternative screening centres?
	**Communication**		
	□ Is there a system to allow early alert of the 1st index case? □ Does the system alert new cluster pickups through the ED? □ Is there a platform for communication with pre-hospital ambulance system? □ Is there a platform for the ED surveillance team to communicate with the infectious disease team in the hospital? (e.g.: Command centre)	□ Are there clear instructions of PPE expectation in the different areas? □ Is there clear communication about staff movement between areas? □ Is there clear communication with patients and relatives about their movement within the different areas?	□ Is the ED able to communicate to the primary healthcare facility about changes in workflows to manage the input? □ Is there a platform to communicate with the hospital inpatient departments? □ Is the ED able to communicate workflow changes to the public?
	**STAFF (Manpower/ Leadership/ Command teams/ Operational teams/ Working teams)**		
	□ Is there a designated ED surveillance team to monitor changes in screening criterion? □ Are there more experienced nursing staff at triage? □ Has security personnel been activated to restrict access into ED during outbreak period?	□ Have vulnerable staff members (e.g. pregnant or immunocompromised) been appropriately re-deployed to minimise exposure. □ Who is responsible for training staff for PPE (ED staff, augmented, ancillary)?	□ Are there plans for augmented manpower at screening area? □ Who trains the augmented staff for screening?
	**SPACE (Infrastructure/ ED spaces/ Ward spaces)**		
	□ Do you have a screening area before triage? □ Are there separate triage areas for patients following screening into high suspect, intermediate suspect and non-suspect categories?	□ Can the spaces be reorganised to reduce exposure & movement of staff?	□ Is the IT system able to capture the patients’ data, to facilitate contact tracing? □ Is there IT support in expanded areas?
	**SUPPLY (PPE/ Cleaning equipment/ Clinical management equipment)**		
	□ Are there any point of care test kits to facilitate screening process?	□ Is there adequate PPE for the different risks areas and does a reliable supply chain exist? □ Are masks provided for patients in waiting areas?	□ Are there standby equipment for areas that need to be expanded?
**ED Throughput**	**SYSTEM**		
	Workflow & protocols		
	□ Is the system able to limit movement of patients between the different suspect areas? □ Is the system able to monitor patients and staff movement between the different suspect areas? □ Is there a workflow for testing in the ED such that the cases can be labelled confirm infected rather than suspected?	□ Is there a strict area demarcation between the different COVID suspect areas? □ Are there regular PPE training & audits? □ Are there handwashing audits or equivalent? □ Is there a workflow for PPE during intubation? □ Is there a workflow for aerosol generating procedures? □ Is there a workflow for the cardiac arrest/ drowsy/ AMS patients?	□ Is there a workflow for high risk suspects with time sensitive conditions (AMI, stroke)? □ Is there a workflow for patients with respiratory symptoms? □ Is there a workflow for dying/palliative patients? □ Is there a workflow for patient intubation? □ Is a rapid diagnostic test being used for the outbreak? □ Is there a workflow with the radiologist to have reports out early? □ Is there a workflow with microbiology for early results? □ Is there a workflow for testing and sending well patients home?
	**Communication**		
	□ Is there a platform to communicate patient workflow and management changes?	□ Are Instructions readily available on donning and doffing of PAPR? □ Is there a platform to communicate with infectious disease specialists within hospital? □ Are there considerations for patients in high risk areas to communicate with staff through portable electronic devices from their negative pressure/ isolation rooms?	□ How is the movement of patients within the ED communicated to ED staff? □ Is there a platform to communicate with radiologists and microbiologists to facilitate investigations done within ED?
	**STAFF (Manpower/ Leadership/ Command teams/ Operational teams/ Working teams)**		
	□ Is there a system to reduce mixing between working teams? (e.g.: modular, team based or staff remaining in their allocated areas for a period of time?)	□ Are there measures to avoid placing the immunocompromised and the pregnant staff in intermediate and high suspect areas? □ Is there a self-monitoring system that allow staff to detect for early signs of infection (e.g.: temperature taking) and report in sick? □ What will be the workflow for the allied healthcare staff in ED? (e.g.: physiotherapy)	□ Is there augmented manpower at the various areas?
	**SPACE (Infrastructure/ ED spaces/ Ward spaces)**		
	□ Is there geographical/ physical segregation between the patients of different risk categories? □ Are there negative pressure rooms in the high suspect areas? □ Is there adequate spacing between the patients in the intermediate and high suspect areas? □ How will radiology spaces be managed?	□ Are there shower facilities for staff? □ Is there adequate space in the pantry for staff to have their meals with physical distancing?	□ Is there a designated path for high and intermediate suspect cases to take to their respective investigation areas (e.g.: CT scan) □ Does the IT system allow the smooth flow of patient information from the screening to their inpatient or community notes?
	**SUPPLY (PPE/ Cleaning equipment/ Clinical management equipment)**		
	□ Are there sufficient equipment for patients to rest on in the various areas without cross contamination risks?	□ Are there disposable/ hospital-based scrubs that staff can wear?	□ Are there alternative equipment for patient management during the outbreak (eg: spacer instead of nebuliser) □ Is there an established supply chain for drugs?
**ED Output**	**SYSTEM**		
	**Workflow & protocols**		
	□ Is there a workflow to determine disposition of the patients?	□ Are there PPE rules for the staff bringing high and intermediate suspect patients being brought to the ward? □ Is there a protocol to clear the route while bringing the patients up to the ward? □ Is there a workflow for interfacility transfer?	□ Is there a workflow to clear inpatient beds during a surge?
	**Communication**		
	□ Is there a method of communicating patients’ ‘suspect status’ when admitting them to the ward? □ Is there a method of communicating patients’ status when doctors from other disciplines review patients in the ED?	□ Is there a good handover regarding patients’ suspect status when sending them to the ward?	□ Is there a platform to communicate with BMU during surges? □ Is there a platform to recall patients after they are d/c? (for contact tracing, information of results)
	**STAFF (Manpower/ Leadership/ Command teams/ Operational teams/ Working teams)**		
	□ Is there adequate manpower in the holding areas?	□ Is the staff transporting the patient trained to wear appropriate PPE? □ Is the security clearing the route trained to wear appropriate PPE?	□ Is there augmented manpower at the holding areas? □ Is the adequate manpower to transport patients? □ Is there adequate manpower to carry out adequate cleaning of the intermediate and high suspect areas?
	**SPACE (Infrastructure/ ED spaces/ Ward spaces)**		
	□ Is there adequate holding areas in the 3 suspect categories?	□ Is there adequate PPE for ancillary staff (Security, admin, porter)? □ Is there supply chain of decontamination substances?	□ Are there spaces that can be opened up during a surge to hold patients awaiting beds? □ Are there sufficient ICU beds and is are there escalation plans when ICU beds are full?
	**SUPPLY (PPE/ Cleaning equipment/ Clinical management equipment)**		
	□ Are there rapid results test kits available to detect the infection and facilitate segregation during disposition? □ Is there adequate partitioning within the ED to keep patients of different risk profiles separated while awaiting disposition?	□ Is there adequate supplies for disposal of contaminated equipment?	□ Is there adequate transport equipment (e.g. wheelchair, trolley)

## Discussion

### Problems posed to the ED at different phases of an outbreak

**Fig. 3:**
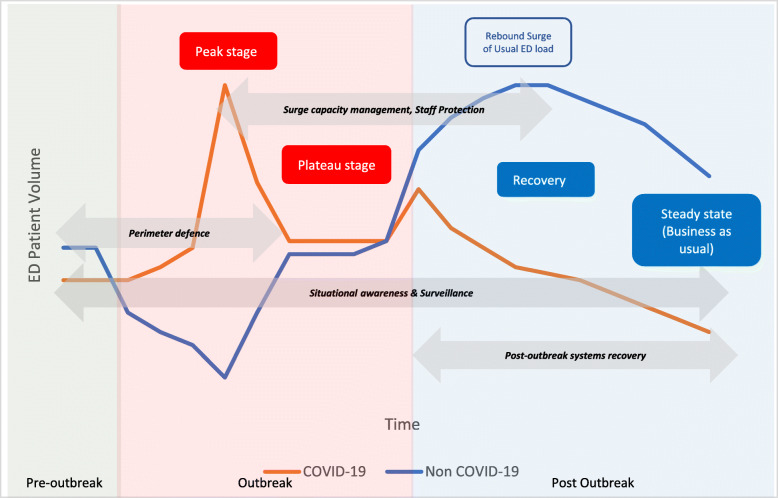
Theoretical model of ED Patient Volumes in a Pandemic

Infectious disease outbreaks pose specific problems to the ED. These problems can be divided into four timeframes as depicted in Fig. 3: pre- outbreak, peak, plateau and post outbreak stages [31]. Before an outbreak, the main issue is with identifying possible index cases seeking treatment in the ED. What should the workflow encompass such that the net for suspects is cast wide without placing a strain on resources? What PPE should the staff at the frontline wear? How should patients enter the ED to mitigate the risk of spreading infection to staff and other patients?

At the peak of the outbreak, there are multiple challenges [43] such as suspect case identification, demand surge, staff safety, patient safety as well as the usual ED workload. The screening of patients, if performed variably, can have hazardous downstream effects such as hospital outbreaks. There needs to be a balance between under and over-triage, keeping in mind local and national guidelines and hospital resources, all of which are dynamic. Not all patients will have typical symptoms, adding to the challenges of designing the ‘perfect’ screening protocol. Availability and performance of test kits may also be an issue, especially early in an epidemic. There can also be difficulties in reconciling public health guidance with the practicalities of ED practice [15]. At the plateau stages, surge capacity will pose a major problem, both at the ED and the hospital level.

### ED workload trend

Figure 3 also depicts the conceptual change in ED workload over time. As the infectious disease workload increases, the usual ED workload may decrease for various reasons. When the outbreak settles and infectious case load decreases, there will be a spike in the normal ED workload. Understanding the ED trend in the local context will help guide the system, space, staff and supplies.

### Post-outbreak recovery

After an outbreak, there will be a surge of acute exacerbations of chronic illnesses, following lockdowns, physical and social isolation and the cancellation of clinic appointments and semi-elective surgeries. The ED should have a system in place to allow recovery to its usual steady state. This includes infrastructure as well as staff. Frontline staff need to be able to function at full capacity even after the outbreak period. At its recovery stage, ED volumes may even be higher due to rebound of patients with complications from neglect of chronic conditions or care-delays that were inevitable during times of crisis.

### The importance of preparedness

Preparation is key to mitigate the disastrous effects of such a pandemic and this preparation needs to be at a National, Hospital and Departmental level. COVID-19 has highlighted the lack of this preparedness can impact lives, strain resources and economic losses. At the frontline, the ED is the defence fortress of the healthcare system. It needs to be in a constant state of preparedness which can be adapted to various pathogens (eg: supply chain of appropriate PPE for highly transmissible pathogens like Ebola).

### ED based vs community-based management

This is an area which warrants further research and guideline development. Based on previous outbreak experiences and the local health system, the public may rush to the ED to be further investigated during an outbreak. If the virus has a high transmission rate with low fatality, perhaps a community approach [20] to screening may reduce the hospital load, allowing them to focus on the critically ill.

### Challenges

In many parts of the world, Emergency Medicine is an under-recognised and under-resourced specialty. This may make the implementation of such a framework challenging. However, the ED or its equivalent is the gateway to the hospital and hence needs to be well established. Alternatively, this framework can be implemented within a department which functions as an ECS.

Another challenge is the cost of applying such a framework. Hence our deliberate attempt to propose a principle-based framework rather than prescribe an actual workflow for other countries. Future work can look at the level of implementation of the checklist with country specific examples.

## Conclusion

Our framework and checklists can be applied to EDs within any health system, including Low- and Middle-Income settings. It focuses on fundamental elements that need to be present for an effective outbreak preparedness system. In preparedness planning, the proposed checklist can be used to guide ED design. The COVID-19 pandemic is forcing us to rethink existing ED models of care, accelerating changes that have always been on our horizon.

## Data Availability

This is not applicable for this paper.

## Abbreviations

ED: Emergency Departments
SARS: Severe Acute Respiratory Syndrome
PPE: Personal Protective Equipment
ECS: Emergency Care Systems
BMU: Bed Management Unit
